# Error in Radar-Derived Soil Moisture due to Roughness Parameterization: An Analysis Based on Synthetical Surface Profiles

**DOI:** 10.3390/s90201067

**Published:** 2009-02-17

**Authors:** Hans Lievens, Hilde Vernieuwe, Jesús Álvarez-Mozos, Bernard De Baets, Niko E.C. Verhoest

**Affiliations:** 1 Laboratory of Hydrology and Water Management, Ghent University, Coupure Links 653, Ghent, Belgium; 2 Department of Applied Mathematics, Biometrics and Process Control, Ghent University, Coupure Links 653, Ghent, Belgium; 3 Department of Projects and Rural Engineering, Public University of Navarre, Pamplona, Spain E-mails: Hans.Lievens@UGent.be; Hilde.Vernieuwe@UGent.be; Jesus.Alvarez@unavarra.es; Bernard.DeBaets@UGent.be; Niko.Verhoest@UGent.be

**Keywords:** SAR, soil moisture, soil roughness parameterization

## Abstract

In the past decades, many studies on soil moisture retrieval from SAR demonstrated a poor correlation between the top layer soil moisture content and observed backscatter coefficients, which mainly has been attributed to difficulties involved in the parameterization of surface roughness. The present paper describes a theoretical study, performed on synthetical surface profiles, which investigates how errors on roughness parameters are introduced by standard measurement techniques, and how they will propagate through the commonly used Integral Equation Model (IEM) into a corresponding soil moisture retrieval error for some of the currently most used SAR configurations. Key aspects influencing the error on the roughness parameterization and consequently on soil moisture retrieval are: the length of the surface profile, the number of profile measurements, the horizontal and vertical accuracy of profile measurements and the removal of trends along profiles. Moreover, it is found that soil moisture retrieval with C-band configuration generally is less sensitive to inaccuracies in roughness parameterization than retrieval with L-band configuration.

## 1. Introduction

Surface soil moisture plays a crucial role in various hydrological and agronomical processes: the top layer moisture content controls the infiltration rate during precipitation events and therefore largely influences the amount of surface runoff, it drives the crop development, and finally, affects the evapo-transpiration rate and thus the micro-climate and -meteorology.

The retrieval of soil moisture content from Synthetic Aperture Radar (SAR) relies on the dependency of the backscattered radar signal on the surface reflection coefficients of the sensed target [1]. These reflection coefficients describe the partitioning of the incoming radar signal into reflected and transmitted energy, and are function of the signal incidence angle, the polarizations of both the incoming and reflected signal and the dielectric constant of the surface target. The high discrepancy between the dielectric constants of respectively dry soil and water allows for assessing the volumetric water content of a wet soil. Dielectric constant values can be converted to soil moisture using several models [e.g., 2, 3].

Apart from soil moisture, the backscattered radar signal shows to be extremely dependent on the roughness state of the sensed surface, in most backscatter models described by the surface root mean square (RMS) height *s*, the correlation length *l* and an autocorrelation function (ACF) [4]. The ACF is mostly chosen to be of exponential or Gaussian type [4], restricting the problem to the derivation of only *s* and *l*. Although the latter roughness parameters are more precisely derived from two-dimensional surface height measurements, e.g. using terrestrial laser or photogrammetric instruments [5], most radar remote sensing studies make use of 1-dimensional surface height measurements for the parameterization of *s* and *l*, for which the current standard procedure is as follows:

1. A series of surface height points (roughness profile) is defined along a 1-dimensional surface transect, mostly sampled by means of meshboard, pin profilometer or laser techniques [6]. Generally, profiles used in practice have a length between 1 m and 4 m [6–9], and the horizontal spacing between height points usually lies between 1 mm [5] and 2 cm [7].
2. From this profile a linear trend is removed to compensate for the possibility that the measurement device was not aligned perfectly parallel to a horizontal reference surface [8].
3. The RMS height *s* can then be calculated as the standard deviation of the series of height points [10], while the correlation length *l* is defined as the horizontal distance over which the correlation between surface height points is larger than 1/*e* [10].
4. As *s* and *l* are extremely variable between different measurements within one agricultural field, both roughness parameters are commonly averaged over a number of profiles, mostly ranging from 3 to 20 [7–9, 11, 12].

This standard parameterization procedure is not absolute: vertical accuracies and horizontal spacings of measured surface points differ for various instruments, causing diverging roughness parameterizations [4, 9]. Moreover, *s* and *l* are subject to a scaling problem, as they generally both increase with increasing profile length [8, 9, 11, 13]. The choice of profile length therefore has a determining influence on the parameterization results. Besides, the assumption of a planar reference surface, justifying the removal of a linear trend from the profile, may only be valid when using short 1-m profiles. Longer profiles, e.g. 4 m in length, often dispose of topographic undulations along the transect and may therefore require the removal of a low-frequency roughness spectrum using a higher-order polynomial.

As briefly summarized above, the parameterization of roughness from profile measurements is characterized by several problems. An extensive literature review on these surface roughness problems is provided by Verhoest *et al* [14]. The present paper focuses on the influence of standard measurement techniques on the parameterization of roughness and its impact on soil moisture retrieval. The remainder of this paper is organized as follows: Section 2. elaborates on the applied soil moisture retrieval technique and its input parameters, Section 3. discusses the sensitivity of this soil moisture retrieval to RMS height and correlation length, further, in Section 4., the generation of synthetical 1-dimensional roughness profiles is explained, and subsequently, a theoretical study on these synthetical profiles is performed in order to assess the influence of roughness parameterization techniques on soil moisture retrieval. The sensitivity of soil moisture retrieval to roughness parameters and the influence of standard roughness parameterization aspects are merely demonstrated on theoretical data, since working with actual SAR data would not allow for a quantitative assessment. The commonly used Integral Equation Model (IEM) [15, 16] is chosen as backscatter model in order to yield similar errors in soil moisture as can be expected in many practical hydrological applications. Finally, conclusions are formulated in Section 5.

## 2. Soil moisture retrieval technique

Many empirical, semi-empirical and theoretical models have been developed to retrieve soil moisture content from the backscattered radar signal. A large number of studies proposed a simple linear empirical relationship between the backscatter coefficient and soil moisture content. Such relationship is easy to apply, however, only valid for a single study site, under the condition that surface roughness remains constant over successive radar acquisitions [e.g., 17, 18]. The mostly used semi-empirical models, developed by Oh *et al.* [19] and Dubois *et al.* [20, 21], are based on a theoretical foundation, however, they still contain model parameters that are derived from experimental data. Conversely, theoretical models present an approximate physical description of wave scattering on rough surfaces. Amongst the mostly used physical approximations are the Small Perturbation Model (SPM) [22], the Kirchhoff Approximations [23] and the IEM [15, 16]. Despite their theoretical foundation, many of these models cannot be applied operationally because of their narrow validity ranges for the majority of natural surfaces. The model with the largest validity range concerning roughness parameters is probably the IEM. Because of this, the IEM has become the most widely used scattering model for bare soil surfaces [24], which gives a sound justification for use in the present theoretical study.

The single scattering approximation of the IEM calculates backscatter coefficients $\sigma_{VV}^{0}$ and $\sigma_{HH}^{0}$, given the dielectric constant *ε* of a bare soil, the radar frequency *f* (GHz), the incidence angle *θ* (°) and roughness parameters: *s* (cm), *l* (cm) and an ACF. Since many authors [e.g., 7, 8, 11, 25] found that for agricultural soils the ACF is well approximated by an exponential function, this type of ACF will be adopted in all further simulations. Based on several experiments, the validity condition of the single scattering approximation of the IEM is often expressed by *ks* < 3 [e.g., 16], with *k* the wave number equal to 2*π/λ* and *λ* the wavelength. In many problems, soil moisture (dielectric constant) needs to be modelled based on observed backscatter coefficients, *i.e.* the IEM should be applied inversely. Several inversion algorithms have been developed, including Look-Up Tables (LUT) [e.g., 26], neural networks [e.g., 27], and the method of least squares [e.g., 28, 29]. Alternatively, the inversion problem can be solved iteratively [e.g., 30], which is preferred in this theoretical study because of its simplicity. To translate the dielectric constant into soil moisture, the four-component dielectric mixing model of Dobson *et al.* [2] is used. Table 1 lists the input parameters for the IEM and the dielectric mixing model used in the remainder of this work. As was also applied by Verhoest *et al.* [31, 32], retrieved moisture contents above 45 vol% are set equal to 45 vol%, whereas moisture contents below 2 vol% are set to 2 vol%, in order to limit the retrieval results to plausible soil moisture contents of real soils.

## 3. Sensitivity of soil moisture retrieval to RMS height and correlation length

**Figure 1.**
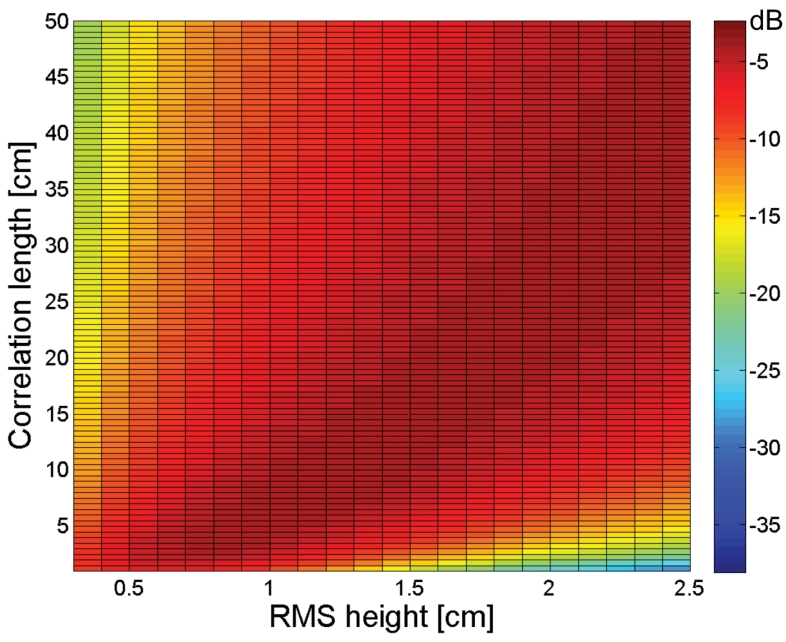

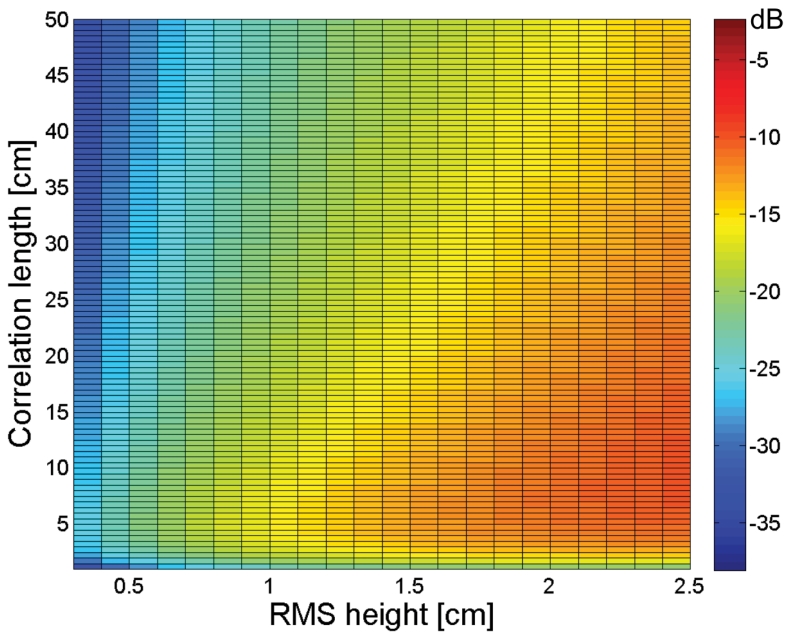
Backscatter coefficients calculated for different values of RMS height and correlation length and a moisture content of 25 vol% for (a) an ASAR VV configuration and (b) a PALSAR HH configuration.

**Figure 2.**
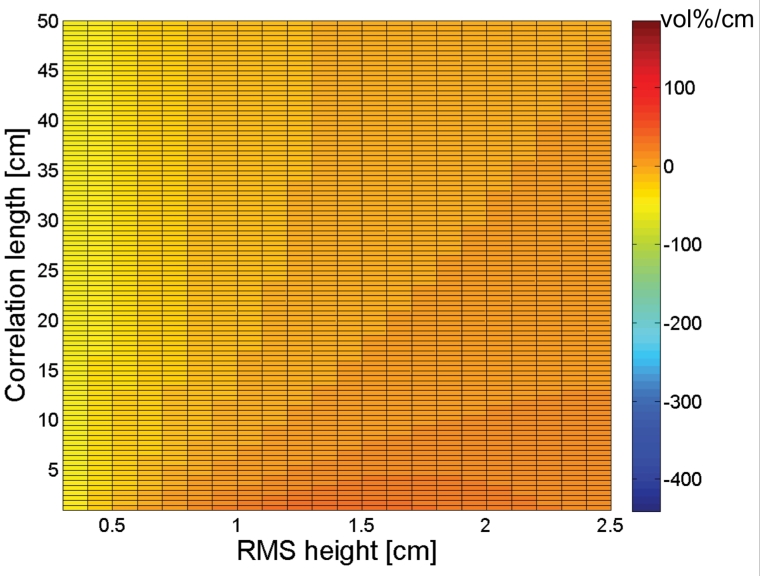

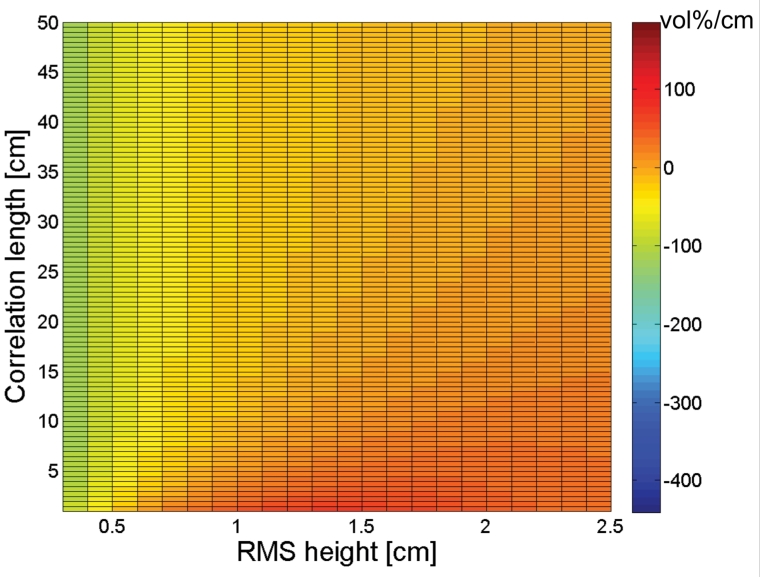

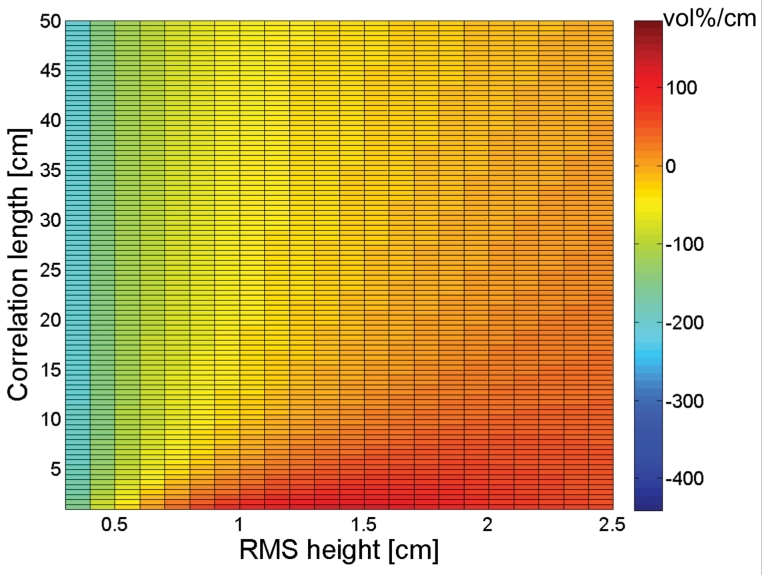

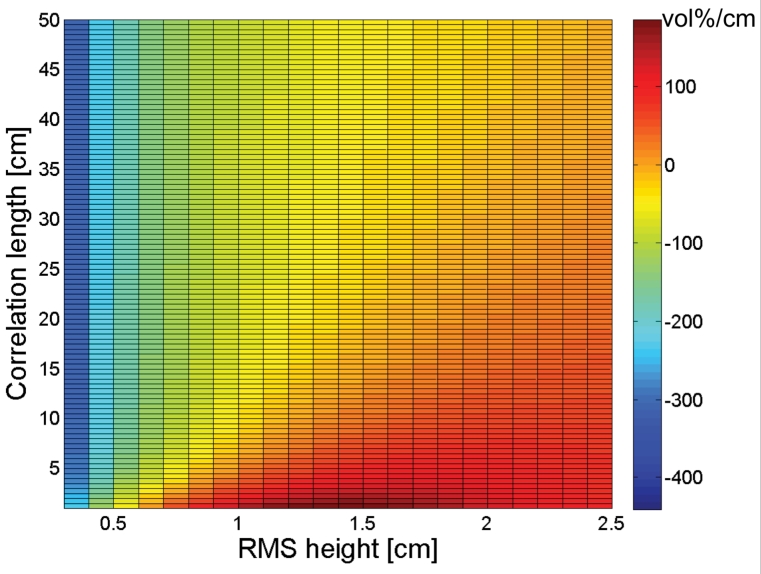
Sensitivity of the soil moisture retrieval to RMS height (vol%/cm) for an ASAR VV configuration and a moisture content of (a) 5 vol%, (b) 15 vol%, (c) 25 vol%, and (d) 35 vol%.

**Figure 3.**
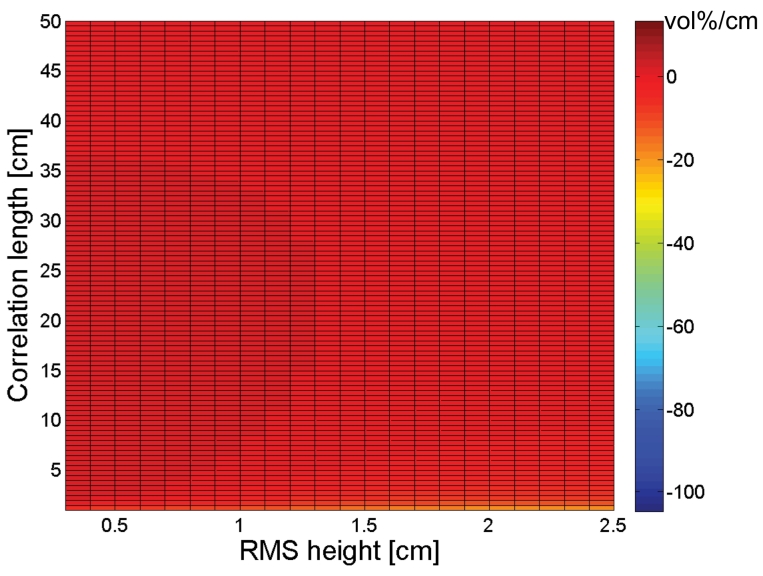

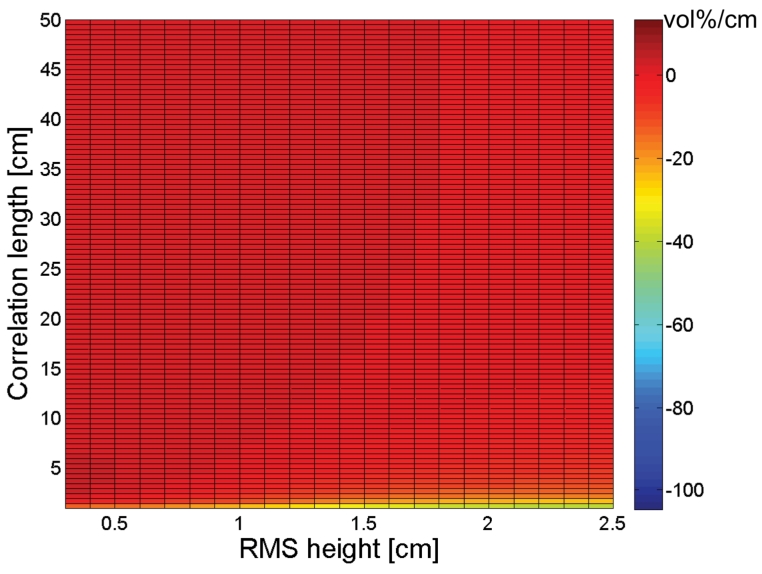

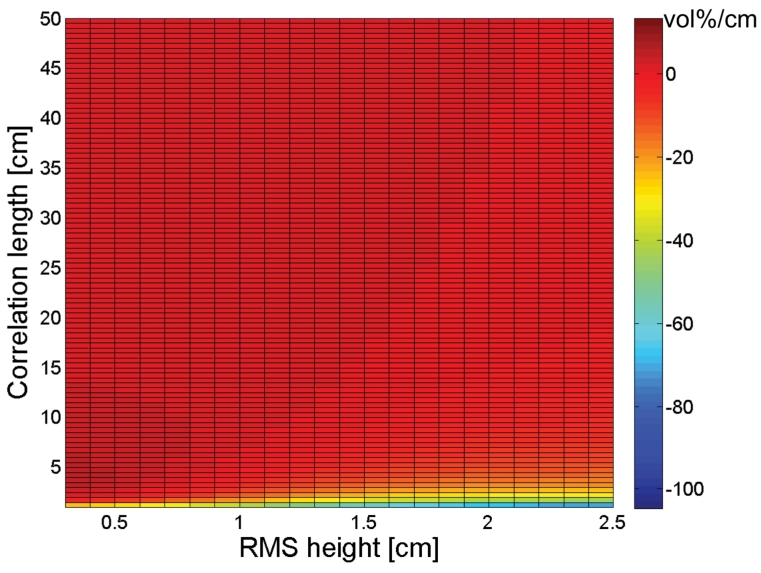

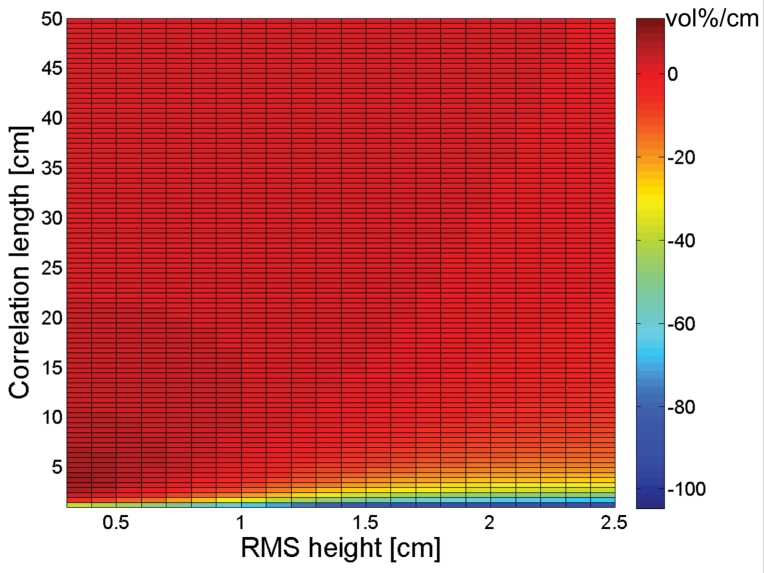
Sensitivity of the soil moisture retrieval to correlation length (vol%/cm) for an ASAR VV configuration and a moisture content of (a) 5vol%, (b) 15vol%, (c) 25vol%, and (d) 35vol%.

**Figure 4.**
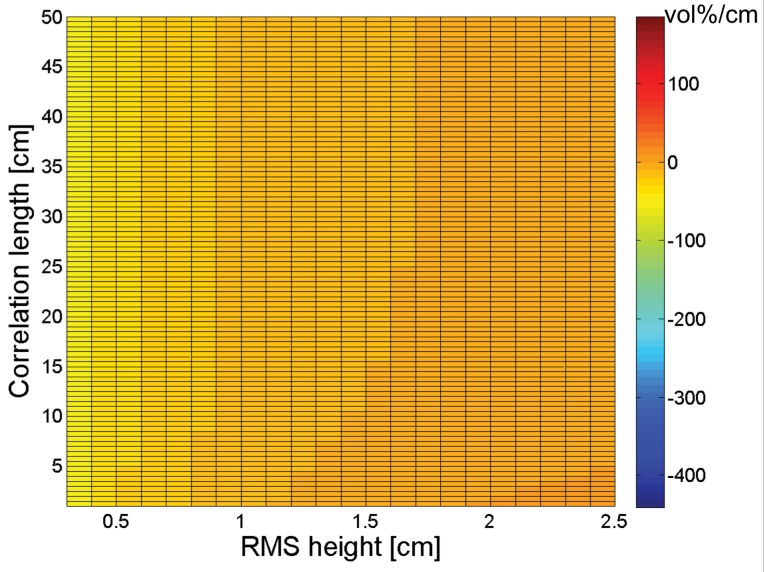

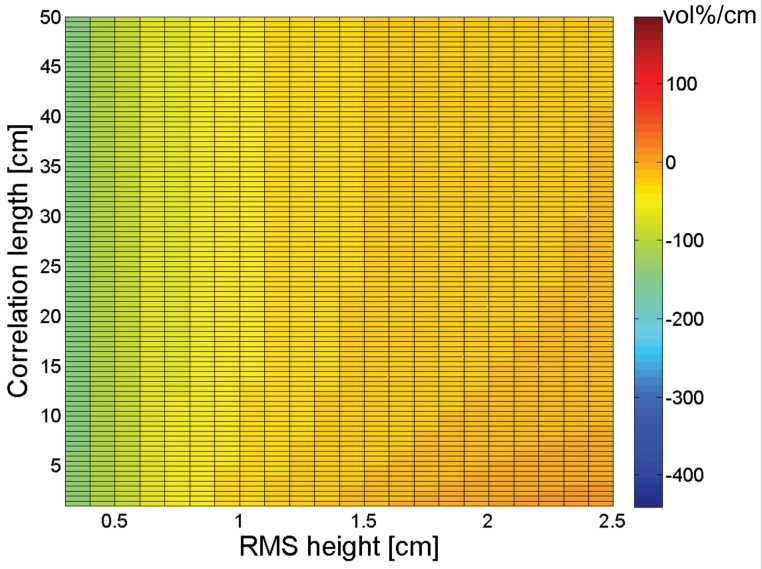

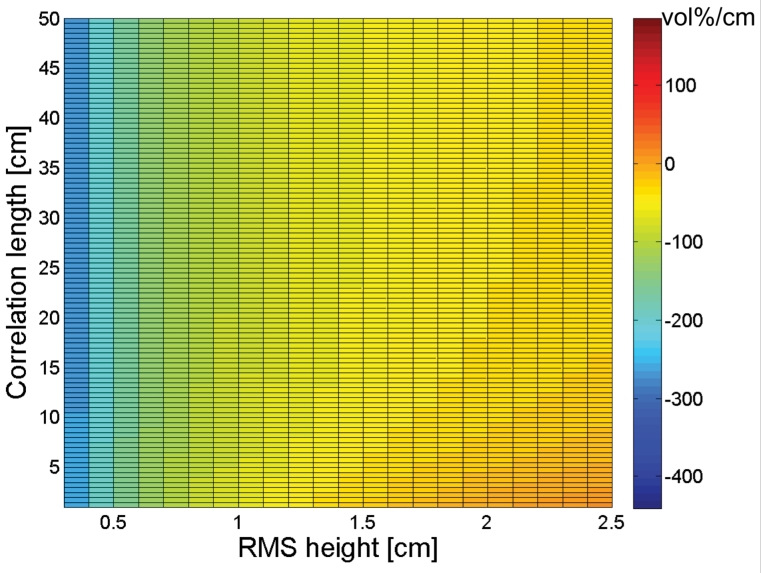

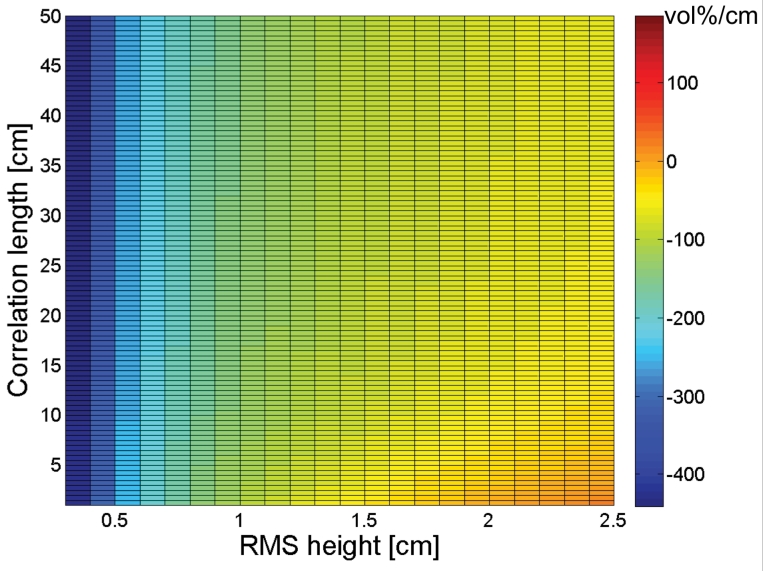
Sensitivity of the soil moisture retrieval to RMS height (vol%/cm) for a PALSAR HH configuration and a moisture content of (a) 5 vol%, (b) 15 vol%, (c) 25 vol%, and (d) 35 vol%.

**Figure 5.**
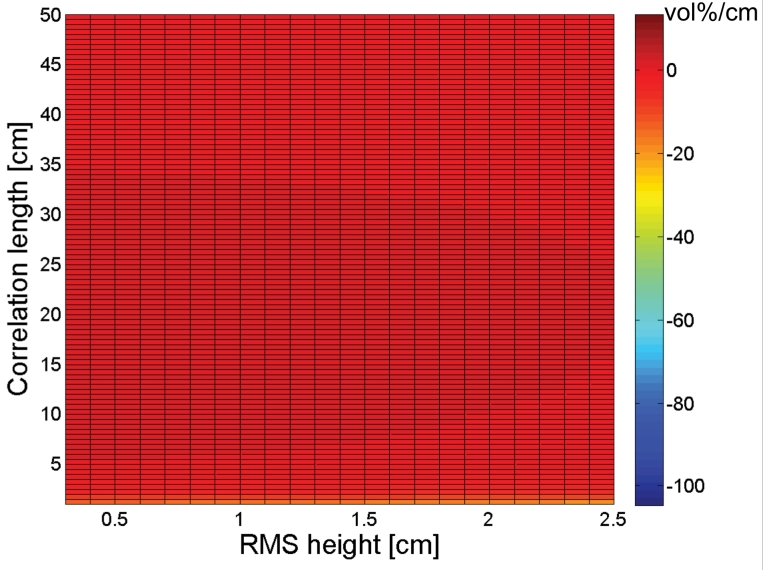

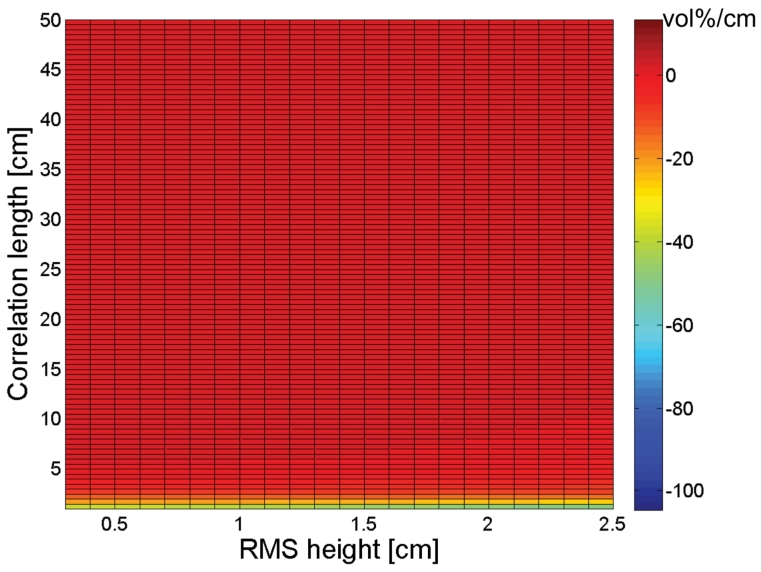

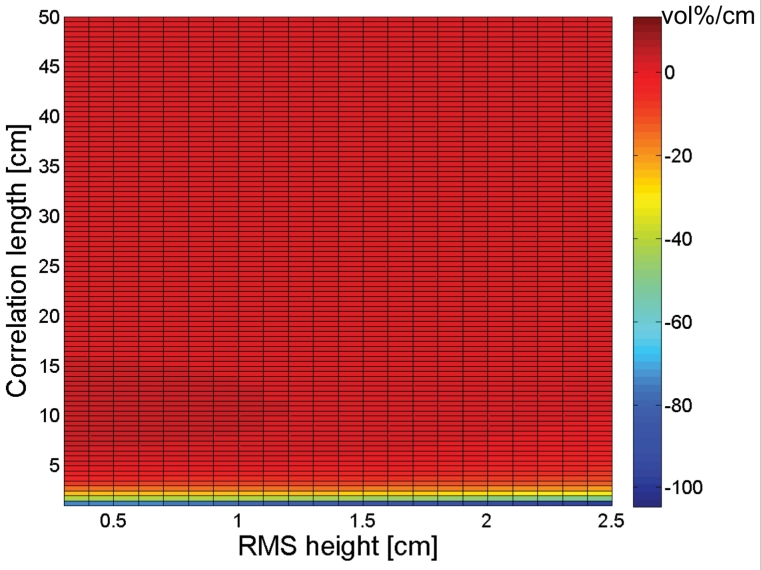

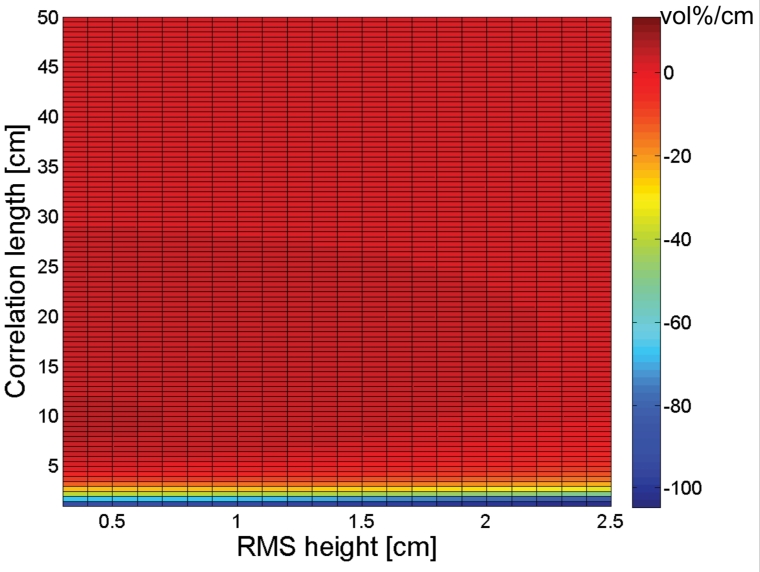
Sensitivity of the soil moisture retrieval to correlation length (vol%/cm) for a PALSAR HH configuration and a moisture content of (a) 5vol%, (b) 15vol%, (c) 25 vol%, and (d) 35 vol%.

As soil roughness largely influences the backscattered signal, one can expect that a correct roughness parameterization is indispensable in order to ensure accurate soil moisture retrieval. To assess the impact of roughness parameterization errors on the soil moisture retrieval, a profound sensitivity analysis is performed using the following experimental setup: for different values of soil moisture (5, 15, 25 and 35 vol%), backscatter coefficients are calculated for *s* &isin; [0.3 cm, 2.5 cm], *l* &isin; [1 cm, 50 cm] and predefined radar configurations, given in Table 1. Figure 1 illustrates the calculated backscatter coefficients for ENVISAT ASAR VV and ALOS PALSAR HH for 25vol% moisture content. As can be seen from this figure, soil roughness may cause a wide range of backscatter coefficients for a soil with a given moisture content. Based on these calculated backscatter coefficients, the sensitivity of soil moisture retrieval to *s* and *l*, respectively, expressed as the gradient of the retrieval surface along the *s* and *l* direction are numerically approximated as:

(1) $$\frac{\partial \text{Mv}}{\partial s}\approx \frac{{\text{Mv}}_{{\text{IEM}}^{-1}}(\sigma_{\text{Mv},s,l}^{0},s+\Delta s,l)-{\text{Mv}}_{{\text{IEM}}^{-1}}(\sigma_{\text{Mv},s,l}^{0},s-\Delta s,l)}{2\Delta s},$$

(2) $$\frac{\partial \text{Mv}}{\partial l}\approx \frac{{\text{Mv}}_{{\text{IEM}}^{-1}}(\sigma_{\text{Mv},s,l}^{0},s,l+\Delta l)-{\text{Mv}}_{{\text{IEM}}^{-1}}(\sigma_{\text{Mv},s,l}^{0},s,l-\Delta l)}{2\Delta l},$$

with $\sigma_{\text{Mv},s,l}^{0}$ the backscatter coefficient calculated with IEM for a given soil moisture content Mv, *s* and *l*, and Δ*s* and Δ/ the discretization steps of 0.01 cm and 0.1 cm, respectively. Finally, it must be stressed that a source of error may be introduced in the calculated backscatter coefficients, presented in Figure 1, due to the use of the single scattering approximation of the IEM. This approximation may cause an underestimation of backscatter coefficients for very rough surfaces that cause a multiple scattering of the SAR signal. Moreover, an underestimation of backscatter coefficients may introduce errors in the presented sensitivity plots in Figures 2 to 5. Therefore, a more cautious interpretation of the sensitivity figures for C-band configuration is adviced in case of large RMS heights, particularly in combination with very small correlation lengths.

Figures 2 and 3 show the sensitivity to *s* and *l*, respectively, for an ASAR VV configuration for the different moisture contents considered. As can be seen from these figures, a small error on the parameterization of *s* influences the soil moisture retrieval much more than a ten times larger error on *l*, which implies that the parameterization of *s* requires a higher accuracy than the parameterization of *l*. Fortunately, Baghdadi *et al.* [33], amongst several others, have shown that generally higher accuracies are obtained for RMS height parameterization than for correlation length parameterization.

As is revealed from Figure 2, the impact of small RMS height errors on soil moisture retrieval increases with increasing moisture content, as was already demonstrated in several publications [e.g., 34]. Also, gradients in the *s* direction are generally negative for small RMS heights together with large correlation lengths, whereas positive gradients are found with large RMS heights and small correlation lengths.

Figure 3 shows that the impact of small correlation length errors on soil moisture retrieval only slightly depends on soil moisture content. An increase in moisture content only causes a substantial increase in sensitivity for very small correlation lengths. However, this effect may be neglected as such small correlation lengths are very unusual for natural surfaces. Conversely to what is found for the sensitivity to *s* in Figure 2, positive gradients generally occur with small RMS heights and large correlation lengths, whereas large RMS heights and small correlation lengths give rise to negative gradients.

Sensitivity plots to *s* and *l* for a PALSAR HH configuration, respectively presented in Figures 4 and 5, show similar trends as found for an ASAR VV configuration. However, comparison of Figures 2 and 4 demonstrates that negative gradients in the *s* direction are more pronounced for a PALSAR HH configuration than for an ASAR VV configuration, whereas the opposite is true for positive gradients. Moreover, a comparison between Figures 3 and 5 reveals that the sensitivity to *l* is less subject to variations in RMS height for a PALSAR HH configuration than for an ASAR VV configuration. Similar sensitivity plots as presented in Figures 2 to 5 are obtained for different combinations of polarization, incidence angle and frequency, and generally show an increased sensitivity for HH polarization compared to VV polarization, and for a higher incidence angle and lower frequency (data not shown).

The sign of the gradients expressed in Figures 2 to 5 allows for assessing whether a given error on the parameterization of *s* or *l* will cause an over- or underestimation of the soil moisture retrieval. If *s* or *l* is underestimated (overestimated), a negative gradient will lead to an overestimation (underestimation) of the soil moisture content, whereas a positive gradient will result in an opposite error. Besides, the magnitude of this gradient provides information on the magnitude of the retrieval error. For example, a gradient of 100 vol%/cm means that a 0.01 cm error on *s* causes a 1 vol% error on soil moisture. However, as the deviation of the parameterized *s* or *l* value from its actual value becomes larger, a first order approximation of the error will be insufficient to describe the actual error made. Moreover, the presented sensitivity figures do not account for simultaneous errors on *s* and *l*.

## 4. Sensitivity of soil moisture retrieval to roughness parameterization techniques

Based on the results of Section 3., one can deduce that even small errors in roughness parameterization, particularly of RMS height, may have a large impact on the soil moisture retrieval. This section further elaborates on the roughness parameterization errors that are associated with standard profile measurement techniques and on the influence of these errors on soil moisture retrieval. To be able to individually address the errors involved with each standard parameterization aspect, synthetical surface profiles are generated. Subsection 4.1. demonstrates the method used to generate such profiles.

The main advantage of synthetical profiles is that they can be designed with a predefined RMS height, correlation length, profile length and spacing between series of height points. Moreover, inaccuracies of the instrument measuring a profile may be easily simulated by adding white noise to each point of the series, and topographic height variations along the profile, which in reality may be caused by either an oblique positioning of the instrument or microrelief, may be introduced by adding a linear or undulating trend.

On the designed profiles, standard parameterization techniques can be applied and evaluated quantitatively. In Subsections 4.2. to 4.6., different experiments are set up to demonstrate respectively the influences of the following parameterization aspects: the profile length, the number of profiles over which *s* and *l* are averaged, the spacing between height points, instrument accuracy and the trend removal technique applied. All experiments are conducted on synthetical profiles with (*s,l*) = (1 cm, 5 cm), (1 cm,40 cm), (2 cm,5 cm) and (2 cm,40 cm), for soil moisture contents of 5, 15, 25 and 35 vol%, and for ASAR and PALSAR HH and VV polarized configurations (see Table 1). Only the most relevant results will be shown.

### 4.1. Generation of synthetical 1-dimensional surface profiles

In order to assess the influence of roughness parameterization techniques on the soil moisture retrieval, a series of synthetical rough surface profiles is generated. The synthetical 1-dimensional profiles are identified using a first order autoregressive model for an exponential ACF:

(3) $$z_{t}=\phi \cdot z_{t-1}+a_{t},$$

with *z_t_* the height at coordinate *t, a_t_* white noise and *φ* a weight factor which can be found from the Yule-Walker equations as [35]:

(4) $$\phi =e^{-\Delta x/l},$$

with Δ*x* the horizontal spacing between height points (cm). Using Equations (3) and (4), a surface profile can be generated with a desired *l*. In order to obtain the desired RMS height, the series is standardized, by subtracting the mean and dividing by the standard deviation, and subsequently multiplied by the desired RMS height.

### 4.2. Profile length

The dependency of roughness parameters on profile length has already been described in depth [e.g., 6, 8, 11, 36]: short profiles generally result in an underestimation of both *s* and *l*, which is more severe for smooth surfaces than for rough surfaces. According to Oh and Kay [11], profile lengths should at least be 40 times the correlation length to obtain the RMS height with a ±10% precision of the mean value, whereas the same accuracy for correlation length only becomes feasible for profile lengths of at least 200 times the correlation length. Although these scaling properties are well known, the impact of using different profile lengths on the soil moisture retrieval has not been reported yet [14].

#### Experimental setup

To assess the influence of profile length on the parameterization of roughness and soil moisture retrieval, extremely long profiles are generated with a Δ*x* of 0.1 cm and predefined (*s,l*) parameters. From such a profile, 500 non-overlapping profiles of different lengths, ranging from 1 m up to 20 m, are sampled, after which the standard roughness parameterization procedure is applied on each of these profiles. As a result, 500 (*s,l*) couples are derived per profile length.

Backscatter coefficients are then calculated for a soil having a specific moisture content and roughness parameters equal to the ones used to generate the extremely long profile. Subsequently, these backscatter coefficients are inverted with IEM^−1^ into soil moisture content, using the roughness parameters from the sampled profiles.

#### Errors on roughness parameterization

**Figure 6.**
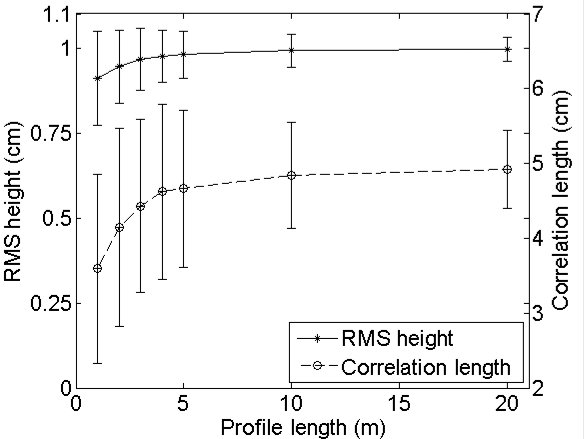

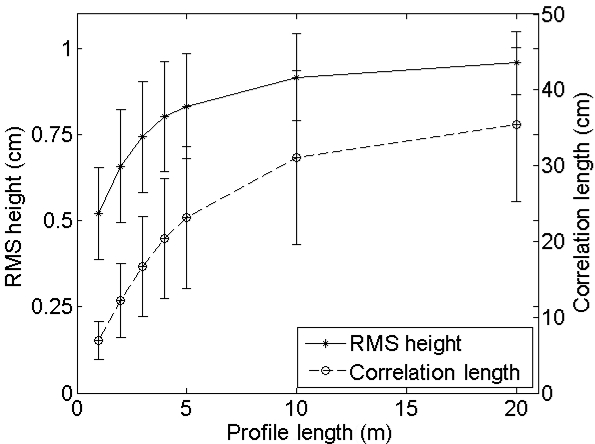
Mean and standard deviations of RMS height and correlation length for different profile lengths, sampled from large profiles with (a) (*s,l*) = (1 cm,5 cm) and (b) (*s,l*) = (1 cm,40 cm).

Figure 6 shows the mean and standard deviations of *s* and *l*, derived from 500 sampled profiles, for different profile lengths. The sampled profiles originate from long profiles with (*s,l*) = (1 cm,5 cm) and (*s,l*) = (1 cm,40 cm). This figure shows for both *s* and *l* a similar scaling behavior as was already reported by Oh and Kay [11], Mattia *et al.* [6] and Callens *et al.* [8]: an increase in profile length leads to an increase of the roughness parameters, which is more pronounced for surfaces characterized by a large correlation length. Similar tests with synthetical profiles of (*s,l*) = (2 cm,5 cm) and (2 cm,40 cm) lead to exactly the same scaling relations as shown in Figure 6 (data not shown), which implies that the scaling behavior of *s* and *l* merely depends on the magnitude of the surface correlation length.

#### Errors on soil moisture retrieval

**Figure 7.**
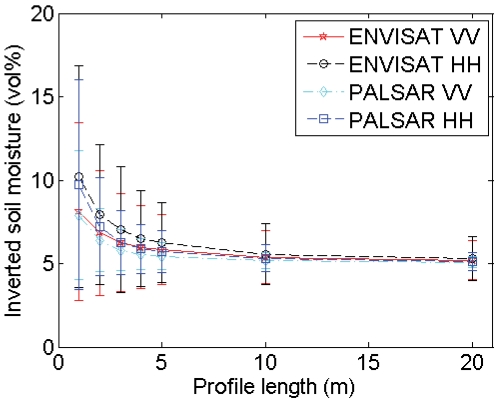

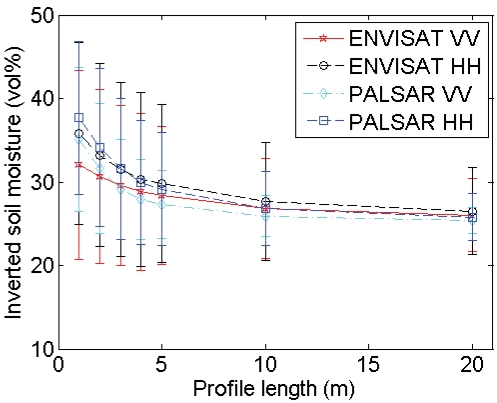

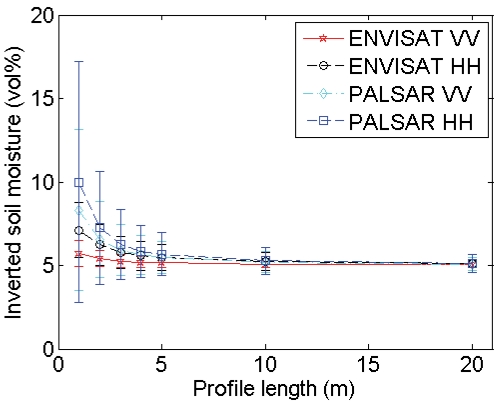

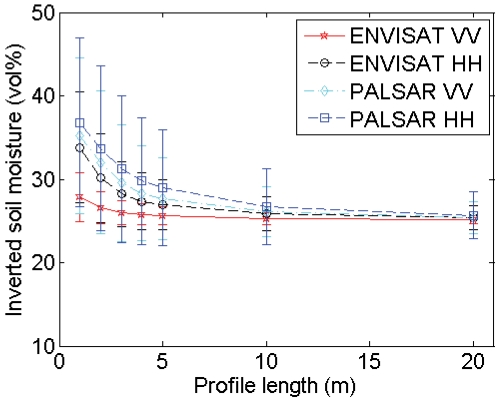

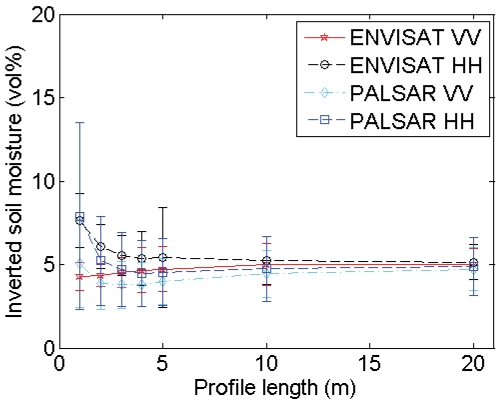

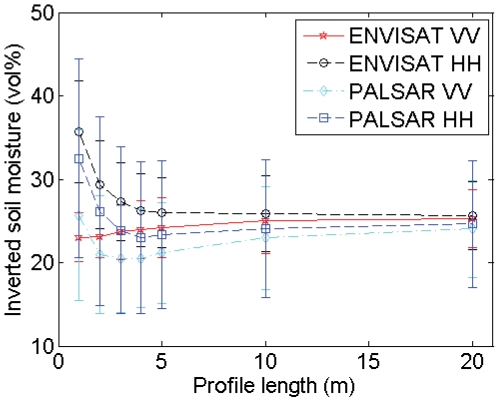

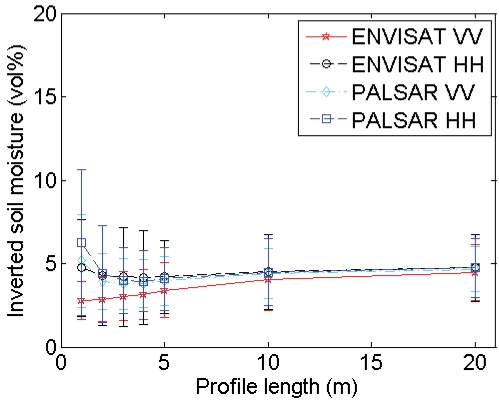

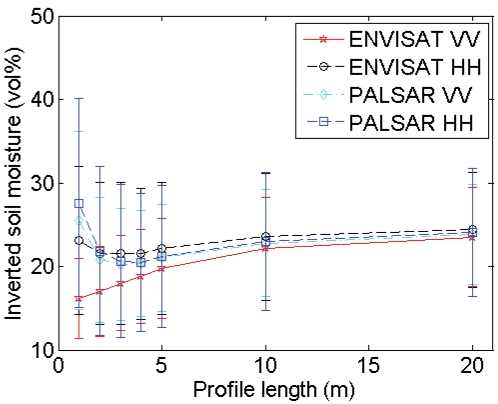
Mean and standard deviations of inverted soil moisture contents for different profile lengths. Inverted soil moisture contents are derived using roughness parameters from sampled profiles, originating from large profiles with (*s,l*) equal to (a), (b) (2 cm,5 cm), (c), (d) (1 cm,5 cm), (e), (f) (2 cm,40 cm) and (g), (h) (1 cm,40 cm), and initial moisture contents of (a), (c), (e), (g) 5 vol% and (b), (d), (f), (h) 25 vol%.

Figure 7 presents the mean and standard deviations of inverted soil moisture contents for different profile lengths and sensor configurations. This figure demonstrates that the inverted soil moisture content may be largely over- or underestimated when using short profiles, particularly for high moisture contents. Moreover, a different behavior is found depending on the roughness state of the surface: the parameterization of *s* and *l* on a rough soil, e.g. (*s,l*) = (2 cm,5 cm), results in overestimated soil moisture contents, whereas opposite errors may be found on smooth surfaces, e.g. characterized by (*s,l*) = (1 cm,40 cm). Overestimations are generally more severe for a PALSAR configuration and HH polarization, whereas underestimations are more pronounced for an ASAR configuration and VV polarization. Finally, for the roughness parameter sets used in the example demonstrated, average retrieval errors for 4-m profiles, which are still feasible to perform in the field [8], are at most about 5 vol%. However, for soil moisture retrievals based on one roughness parameter set, extremely large errors are found, as illustrated by the standard deviations shown in Figure 7.

A rough estimate of the soil moisture retrieval error for a surface with given moisture content, roughness state and a certain sensor configuration may also be deduced from Figures 2 to 5. As an example, Figure 6 shows that a 4-m profile, used on a surface with (*s,l*) = (1 cm,40 cm), on average results in (*s,l*) = (0.8 cm,20 cm). From Figures 2 and 3, it can be seen that, for a moisture content of 25 vol% and an ASAR VV configuration, gradients in the *s* and *l* direction are respectively -50 vol% and 0.7 vol%. Based on these gradients and the errors on *s* and *l*, a retrieval error of -4 vol% is found, which approximately resembles the error demonstrated in Figure 7 (h) at 4-m profile length.

In this experimental setup, it was assumed that an optimal parameterization of roughness requires an extremely large profile, resulting in precise asymptotic roughness parameters (see Figure 6). However, if a shorter initial profile would have been used for sampling, the scaling behavior of *s* and *l* would have been similar, however, resulting in different soil moisture contents. Unfortunately, it is currently not yet known on which spatial scale roughness parameters need to be defined in order to be most appropriate for describing scattering on rough surfaces. The above example may therefore only be seen as an illustration of the errors that can be expected when using different profile lengths for the same problem.

### 4.3. Number of profile measurements

According to Bryant *et al.* [9], the surface RMS height needs to be averaged over at least twenty 3-m profiles in order to be representative. Using 2-m profiles with correlation lengths between 2 and 20 cm, Baghdadi *et al.* [12] demonstrated that by averaging roughness values over 10 profiles, the RMS height can be derived with a precision better than ±5%, whereas the precision for correlation length ranges from ±5% to ±15%. According to Davidson *et al.* [7], Callens *et al.* [8] and Oh and Kay [11], this variability decreases with increasing profile length. In this section, a theoretical experiment is set up to assess the minimum number of profiles that is needed to obtain roughness parameters with a precision of ±10% of their mean value, and subsequently, to investigate the effect of using averaged roughness parameters for soil moisture retrieval.

#### Experimental setup

A field experiment is simulated in which roughness parameters are determined by averaging *n* profiles of a certain length (*n* ranging from 1 to 20). In order to assess the variability of the determined average roughness parameters, the same procedure is repeated 1000 times, *i.e.* if *n* = 4, in total 4000 profiles of a certain length are sampled from an extremely large synthetical roughness profile. Based on the obtained series consisting of 1000 averaged roughness parameters for different numbers of profiles *n*, one can find the number *n* for which the standard deviation of *s* or *l* is less than 10% of the mean. The experiment is performed for sampled profiles with lengths ranging from 1m up to 20 m.

Next, backscatter coefficients are calculated for given moisture contents, sensor configurations and the roughness parameters used to generate the extremely large profiles, and are subsequently inverted with IEM^−1^ into soil moisture content, using the series of averaged roughness parameters from sampled profiles. Only 4-m profiles are considered in this part of the experiment, as these are frequently used in practice.

#### Errors on roughness parameterization

**Figure 8.**
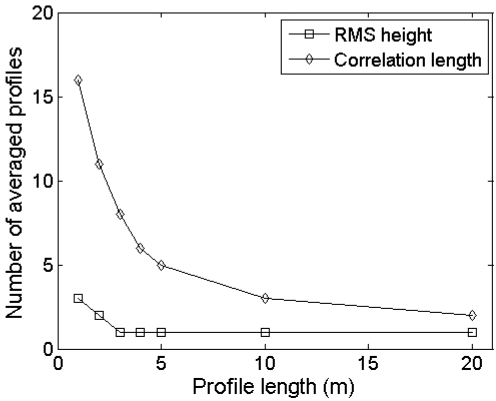

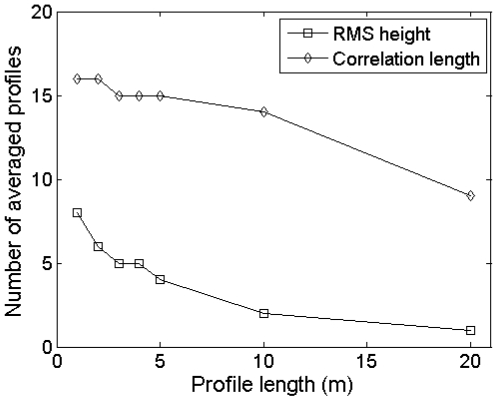
Number of profiles required to obtain a standard deviation of RMS height or correlation length less than 10% of the mean for different profile lengths. Sampled profiles originate from large profiles with (*s,l*) equal to (a) (1 cm,5 cm) and (b) (1 cm,40 cm).

Figure 8 shows the number of profiles that is required to obtain a standard deviation on RMS height or correlation length less than 10% of the mean, for different sampled profile lengths from large profiles with (*s,l*) = (1 cm,5 cm) and (*s,l*) = (1 cm,40 cm). As can be seen in this figure, the required number of profiles decreases with increasing profile length. Moreover, as surfaces with a larger correlation length show more variability in roughness parameterization (see Figure 6), the required number of profiles consequently increases with an increase of *l*. Similar tests on surfaces with (*s,l*) = (2 cm,5 cm) and (*s,l*) = (2 cm,40 cm) reveal that this number is not influenced by *s*, as the same plots as those presented in Figure 8 are obtained (data not shown).

#### Errors on soil moisture retrieval

**Figure 9.**
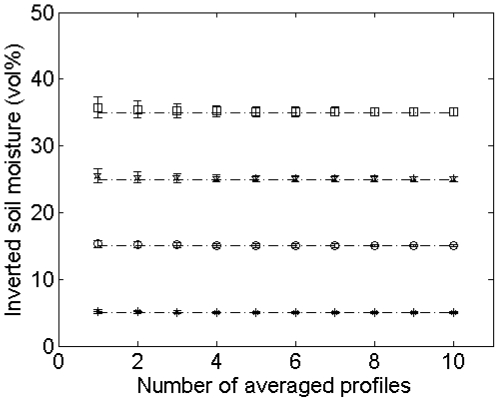

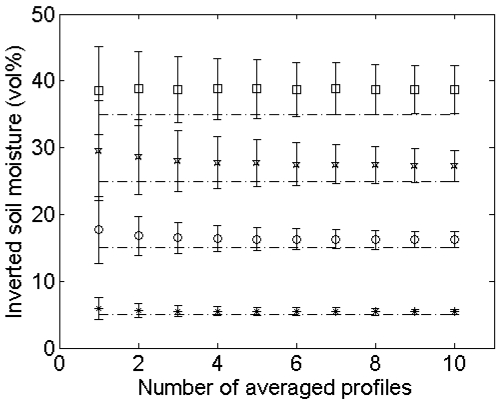

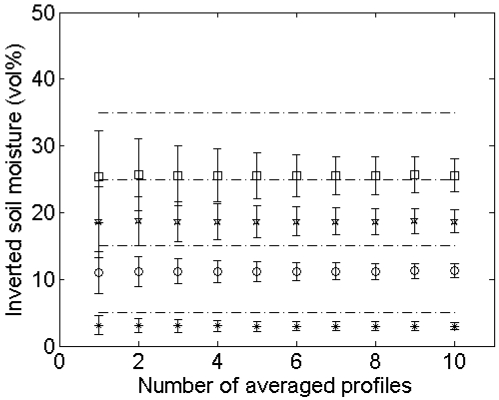

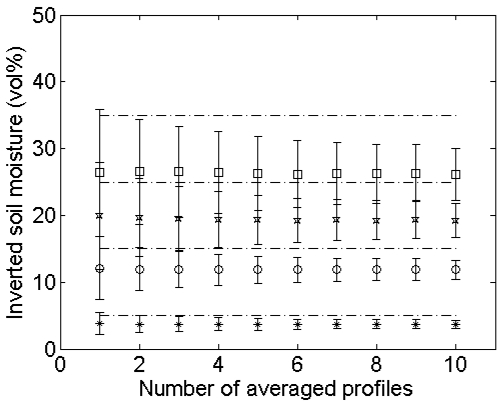
Mean and standard deviations of inverted soil moisture contents for different numbers of profiles used. Inverted soil moisture contents are derived using roughness parameter series from sampled 4-m profiles, originating from large profiles with (*s,l*) equal to (a), (b) (1 cm,5 cm) and (c), (d) (1cm,40 cm), and for (a), (c) ASAR VV and (b), (d) PALSAR HH. Considered initial moisture contents are 5 vol% (crosses), 15 vol% (circles), 25 vol% (stars) and 35 vol% (squares).

Analysis of the soil moisture retrieval error for sampled 4-m profiles from surfaces with (*s,l*) = (1 cm,5 cm) and (*s,l*) = (1cm,40 cm) and ASAR VV and PALSAR HH configurations (see Table 1), as illustrated in Figure 9, reveals that an increase in the number of profiles used to average roughness parameters only causes a moderate decrease of the standard deviation of inverted soil moisture contents. Conversely, the sensor configuration, soil moisture content and surface roughness state have a much higher impact on this standard deviation. In general, larger standard deviations are obtained for higher moisture contents, PALSAR HH configuration and surfaces with larger correlation length.

As can be seen in Figure 9, the mean inverted moisture content can be strongly biased, particularly for high moisture contents and roughness parameters (*s,l*) = (1 cm,40 cm), which is due to the scaling problem of roughness as discussed in Section 4.2. Similar tests for an ASAR HH and a PALSAR VV configuration show intermediate results compared to ASAR VV and PALSAR HH configurations (data not shown).

### 4.4. Spacing between height points

The horizontal spacing between discrete height observations along the profile is mostly defined by the instrument used. For laser devices, this spacing commonly ranges between 1 mm [5] and 5 mm [7], whereas for pin profilometers, horizontal distances between adjacent height measurements ranging from 2 mm [5] up to 2 cm [37] have been reported. According to Ulaby *et al.* [38], a spacing of 1/10 of the wavelength of the SAR signal is recommended. However, according to Ogilvy [4], the horizontal spacing should not exceed 0.1 times the correlation length for accurate parameterization of roughness. Larger spacings cause a change in slope of the ACF around zero, as the high-frequency component (height deviations over very small horizontal distances) is lost. Therefore, larger spacings may cause the ACF to resemble a Gaussian function, whereas in reality the function has an exponential shape. Such false interpretation may lead to large retrieval errors.

#### Experimental setup

To assess the impact of the horizontal spacing on the roughness parameterization and soil moisture retrieval, an experiment is set up in which ten 4-m profiles with given set of (*s,l*) parameters are generated with a spacing of 1 mm. These profiles are then resampled to spacings of 2, 5, 10 and 15 mm, after which RMS heights and correlation lengths are calculated.

Subsequently, these roughness parameters are used for soil moisture retrieval from backscatter coefficients, obtained for given soil moisture contents and the roughness parameters defined at 1-mm spacing. Finally, the effect of a misinterpretation of the ACF on the soil moisture retrieval is investigated using the calculated roughness data from resampled profiles with 15-mm spacing.

#### Errors on roughness parameterization

Mean and standard deviations of roughness parameters calculated from ten resampled profiles are shown in Table 2. In general, it can be found that an increase in horizontal spacing causes a decrease in RMS height and an increase in correlation length, which are more pronounced for surfaces with small correlation lengths.

#### Errors on soil moisture retrieval

**Figure 10.**
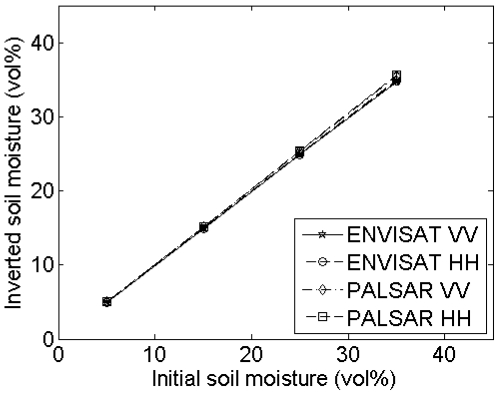

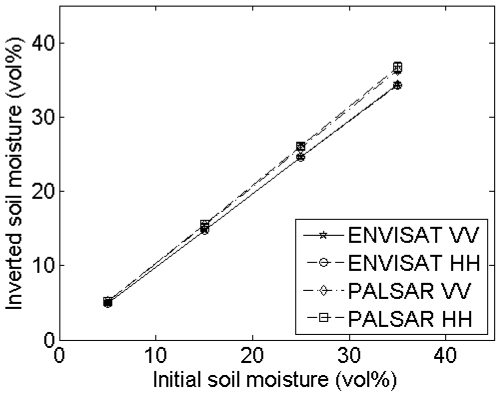

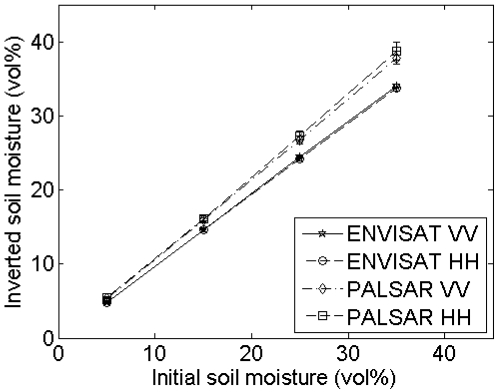

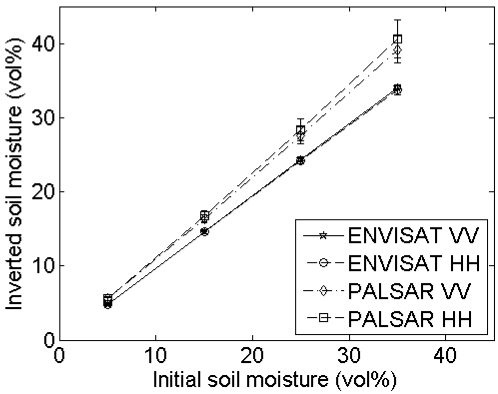
Mean and standard deviations of inverted soil moisture contents for different horizontal spacings used in the parameterization of roughness from 4-m profiles with (*s,l*) = (1 cm,5 cm). Considered spacings are (a) 2mm, (b) 5mm, (c) 10mm and (d) 15mm.

**Figure 11.**
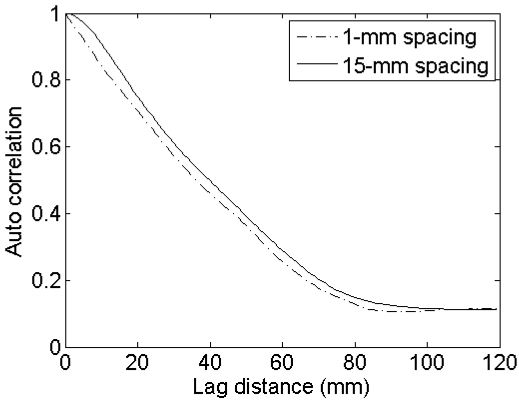
Autocorrelation functions derived for the same roughness profile with (*s,l*) = (1 cm,5 cm), sampled with a spacing of respectively 1mm and 15 mm.

**Figure 12.**
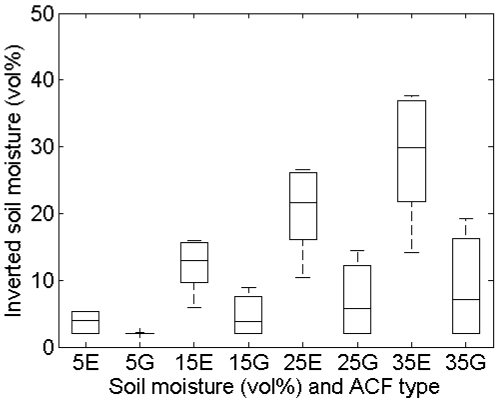

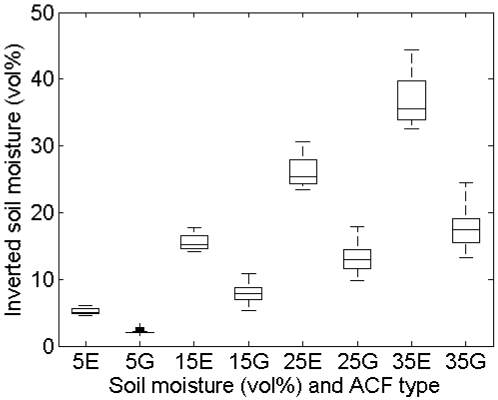

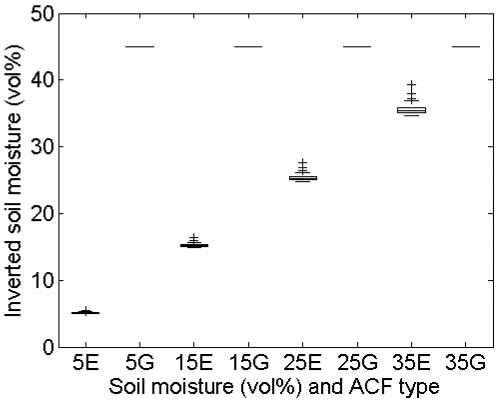

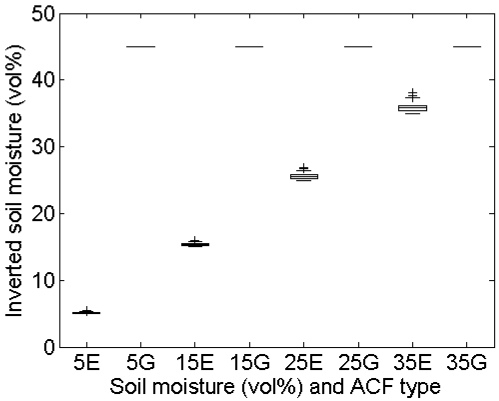
Boxplots of inverted soil moisture contents, calculated using roughness parameters from resampled profiles with 15-mm spacing, and exponential (E) and Gaussian (G) autocorrelation functions, for initial moisture contents of 5, 15, 25 and 35 vol%, and all defined sensor configurations, for profiles with (*s,l*) equal to (a) (2 cm,5 cm), (b) (1 cm,5 cm), (c) (2cm,40 cm) and (d) (1cm,40 cm).

Mean and standard deviations of inverted soil moisture contents for different horizontal spacings and sensor configurations (Table 1) and a surface with (*s,l*) = (1 cm,5 cm) are shown in Figure 10. This figure illustrates that larger spacings give rise to larger retrieval errors, being more pronounced for higher moisture contents. Smaller retrieval errors are found for a smooth surface with (*s,l*) = (1 cm,40 cm), whereas larger errors are obtained for a rough surface with (*s,l*) = (2 cm,5 cm) (data not shown). Finally, errors involved with a PALSAR configuration are generally larger than errors obtained for an ASAR configuration. The latter results may also be derived using the data presented in Table 2 and the sensitivity plots shown in Figures 2 to 5. The effect of the horizontal spacing on the ACF is demonstrated in Figure 11 for profiles with a spacing of 1 mm and 15 mm. It is clear that a steeper slope at the origin is encountered for the profile with 1-mm spacing than for the profile with 15-mm spacing, illustrating the loss of the high-frequency roughness component with an increase in distance between measurement points. If the ACF is therefore assumed to be Gaussian, whereas in reality the profile is characterized by an exponential ACF, extreme retrieval errors covering the entire range of soil moisture content may be found, as illustrated by the boxplots in Figure 12. As is revealed from these boxplots, soil moisture retrieval using a Gaussian ACF leads to severe underestimations in case of a rough surface, and conversely, severe overestimations in case of a smooth surface. Finally, standard deviations of the retrieved soil moisture contents are much larger for rough surfaces than for smooth surfaces, as for rough surfaces the retrieval results are more diverging for the different sensor configurations considered.

### 4.5. Instrument accuracy

The accuracy of instruments that measure discrete surface height points varies from less than 1 mm for non-contact techniques, such as laser profilometers, up to 2.5 mm for instruments that require a destructive contact with the surface, e.g. meshboards and pin profilometers [5]. In case a meshboard is used, the accuracy may additionally decay because of the digitization process needed to outline the surface [14]. D'Haese *et al.* [39] digitized the same profile ten times and found a coefficient of variation of 1.7% on RMS height and 6.5% on correlation length, with an average (*s,l*) = (0.96 cm,10.2 cm). The same profile, digitized by 12 different people, leads to a coefficient of variation of 4.52% and 4.51% for respectively RMS height and correlation length. In case a pin profilometer is used, the digitization can be processed electronically and its influence on RMS height becomes negligible [40].

#### Experimental setup

To assess the effect of the instrument accuracy on the roughness parameterization and consequently soil moisture retrieval, the following experiment is carried out: a noisy signal, uniformly distributed in [−*a,a*], with *a* the accuracy assumed (1mm, 2mm or 5 mm), is added to ten 4-m wide synthetical roughness profiles with a predefined *s* and *l*, after which the roughness parameters are determined.

The calculated roughness parameters may then be used for inversion of backscatter coefficients obtained with given moisture contents and the predefined roughness parameters from profiles without added noise signal.

#### Errors on roughness parameterization

The Root Mean Square Errors (RMSE) between roughness parameters calculated on the noisy profiles and predefined roughness parameters are presented in Table 3. As is revealed from this table, errors are marginal for inaccuracies up to 2 mm, typically involved with laser and pin profilometer measurements. Conversely, large inaccuracies up to 5 mm, which are possible in case of using meshboards and manual digitization, may result in an RMSE up to about 0.04 cm and 3.00 cm, respectively for *s* and *l*, as found for a smooth surface with (*s,l*) = (1 cm,40 cm).

#### Errors on soil moisture retrieval

Soil moisture retrieval errors due to an inaccurate roughness parameterization are presented in Table 4 for a PALSAR HH configuration (ASAR and VV configurations both lead to smaller errors). In general, retrieval errors are less than 2 vol% for instruments with a noise level smaller than 2 mm. Moreover, the retrieval error increases with an increase in moisture content and is dependent on the surface roughness, with the largest errors for the surface of (*s,l*) = (1 cm,40 cm). Instruments with a noise level of 5 mm may result in errors ranging from ±0.5 vol% for dry and rough fields up to ±8 vol% for wet and smooth fields. Given these results, a cautious use of low resolution instruments is advised, since their roughness parameterization may introduce large retrieval errors.

### 4.6. Trend removal

The standard procedure for roughness parameterization includes the removal of a linear trend from a profile to compensate for the fact that the profile transect may be slightly tilted with respect to a horizontal reference surface. However, in case a field shows a slightly undulating surface, corresponding to roughness at very low frequency, it is currently not known whether or not such low-frequency component should be removed from the profile in order to precisely measure the roughness spectrum as it is sensed by a radar signal, particularly for high-frequency radar (e.g. at C-band). As argued by Ulaby *et al.* [10], only the high-frequency component should be maintained for the parameterization of roughness, whereas a low-frequency roughness component should be included directly in the backscatter model. According to Callens *et al.* [8], the use of long profiles from 4 m onwards on slightly undulating fields requires the removal of a second- or third-order polynomial. Alternatively, Bryant *et al.* [9] introduced piecewise 1-m linear trend removal over longer profiles. The present section is not aiming at investigating which type of trend should be removed from long surface profiles in order to characterize the roughness spectrum as encountered by the radar signal, but rather, it intends to assess the influence of using different trend removal techniques on the parameterization of roughness and the soil moisture retrieval.

#### Experimental setup

**Figure 13.**
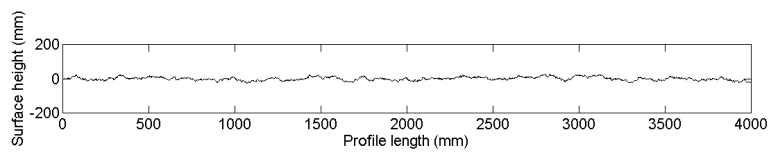

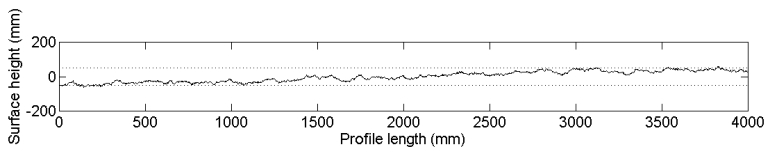

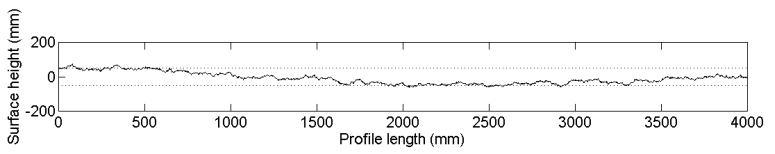
(a) Original simulated 4-m roughness profile with (*s,l*) = (1 cm,5 cm) and a horizontal spacing of 1 mm, added to (b) a linear trend with slope of 0.025 m/m, and (c) a cosine trend with a wavelength of 5m and an amplitude of 5 cm

To assess the impact of commonly used detrending techniques on roughness parameterization and soil moisture retrieval, ten 4-m profiles are generated with predefined (*s,l*). Furthermore, two trend surfaces are generated: (1) a planar surface with a slope of 0.025 m/m, and (2) a slightly undulating surface simulated by a cosine function with wavelength of 5 m and amplitude of 5 cm, which in reality will not be seen as a major deviation from a planar surface. Next, these trend surfaces are added to the ten 4-m profiles. Figure 13 shows examples of an original 4-m profile, a linear trended profile and a cosine trended profile. Subsequently, trends are removed by subtracting a 4-m linear function, piecewise 1-m linear functions, and second- and third-order polynomials, after which the roughness parameters are calculated.

The derived roughness parameter sets are then used to invert backscatter coefficients obtained for the predefined (*s,l*) parameters from the original 4-m profiles and predefined soil moisture contents.

#### Errors on roughness parameterization

Table 5 shows the mean and standard deviations (between brackets) of the calculated roughness parameters after detrending of ten linear and cosine trended profiles using the techniques considered. Analysis of this table reveals that even 4-m linear detrending of linear trended profiles may lead to large errors in the parameterization of *s* and *l*, with both parameters being underestimated. Note that the linear de-trended profile may differ from the initial non-trended profile. This can be explained by the fact that the initially generated 4-m profile may show a non-flat first order regression, caused by the autocorrelated nature and randomness of the profile generation process. Higher-order polynomials clearly delete some roughness on linear profiles, resulting in lower *s* values, however, the largest errors are found with piecewise linear detrending over 1-m subprofiles. On cosine trended profiles, most precise roughness parameters are obtained using higher-order polynomials. Linear detrending causes *s* and *l* to be overestimated, whereas piecewise detrending still leads to a large underestimation of both roughness parameters.

#### Errors on soil moisture retrieval

**Figure 14.**
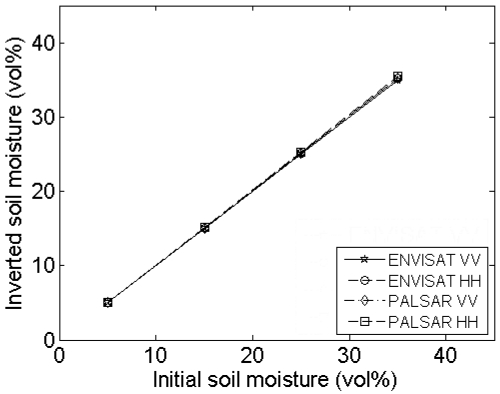

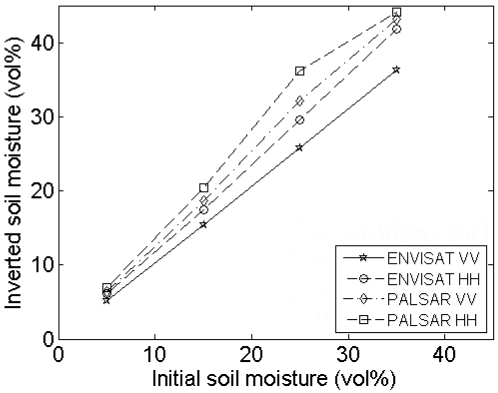

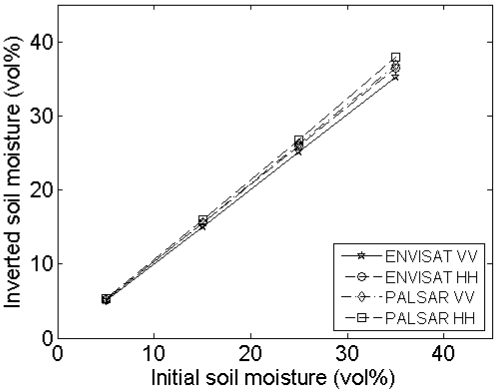

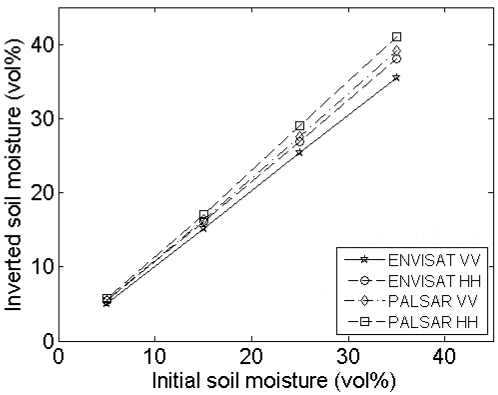
Mean inverted soil moisture contents for different radar configurations, using roughness parameters obtained after (a) linear detrending over the 4-m profile, (b) piecewise 1-m detrending, (c) second-order polynomial detrending and (d) third-order polynomial de-trending of 10 synthetical roughness profiles of (*s,l*) = (1 cm,5 cm) with added linear trend.

**Figure 15.**
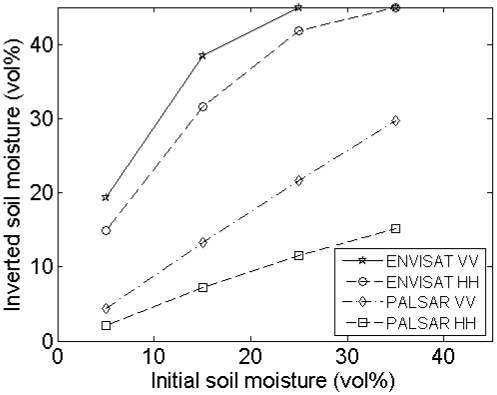

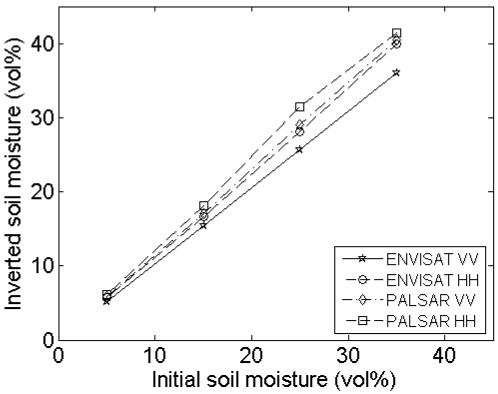

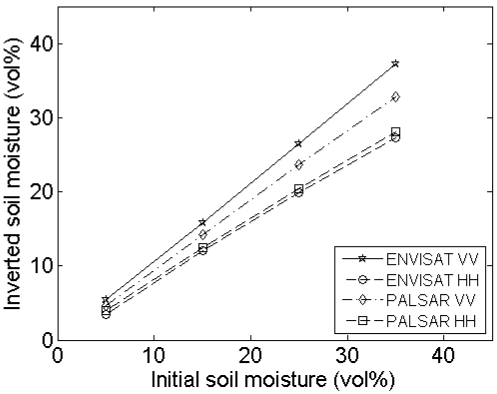

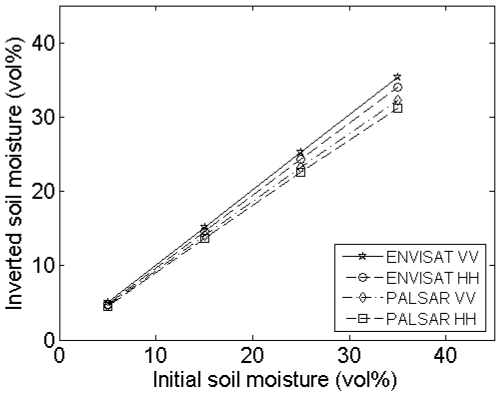
Mean inverted soil moisture contents for different radar configurations, using roughness parameters obtained after (a) linear detrending over the 4-m profile, (b) piecewise 1-m detrending, (c) second-order polynomial detrending and (d) third-order polynomial de-trending of 10 synthetical roughness profiles of (*s,l*) = (1 cm,5 cm) with added cosine trend.

Figures 14 and 15 respectively demonstrate inverted soil moisture contents for linear and cosine trended surfaces with (*s,l*) = (1 cm,5 cm). Based on these figures, 4-m linear detrending is found to be superior in case the profile is characterized by a linear trend, however, if applied on cosine trended profiles, retrieval errors up to ±25 vol% may be expected. Moreover, in case of 4-m linear detrending, retrieved soil moisture contents are largely diverging for the different sensor configurations considered. Conversely, soil moisture errors involved with piecewise linear detrending and higher-order polynomial detrending are relatively low, despite the large errors found in the roughness parameterization. These low errors may probably be attributed to the fact that *s* and *l* are generally biased in the same direction, whereas gradients in the *s* and *l* directions have opposite signs for most parameter combinations, as illustrated in Figures 2 to 5.

Similar tests on profiles with (*s,l*) = (2 cm,5 cm) show analogous results. However, tests on profiles with (*s,l*) = (1 cm,40 cm) reveal that, using a second-order polynomial, inverted soil moisture contents may be underestimated up to approximately 15 vol%, whereas third-order polynomial detrending results in less severe underestimations of only 7.5 vol% (data not shown) and therefore appears to be the most appropriate simple technique for the removal of undulating trends.

Based on this experiment, it cannot be decided whether or not undulations along profiles should be removed prior to roughness parameterization. However, it can be concluded that soil moisture retrieval results may be very different in case undulations are removed through non-linear detrending than in case they are maintained, *i.e.* with linear detrending. Moreover, third-order polynomial detrending shows to be more appropriate for the removal of such undulations than second-order detrending and piecewise 1-m linear detrending. Nevertheless, as even this technique may still lead to substantial errors, future research definitely needs to explore more advanced trend removal techniques, such as techniques based on spectral analysis.

## 5. Conclusions

Correct surface roughness parameters are of extreme importance in order to accurately retrieve soil moisture from SAR. A sensitivity study of the soil moisture retrieval to RMS height and correlation length reveals that small errors on RMS height generally more affect the soil moisture retrieval than ten times larger errors on correlation length. Therefore, RMS height parameterization requires a higher accuracy than the parameterization of the correlation length. Besides, sensitivity surfaces of respectively *s* and *l* generally show opposite trends, causing soil moisture retrieval errors to be partially cancelled out if both roughness parameters are biased in the same direction. Finally, sensing with an L-band HH configuration increases the sensitivity to roughness compared to a C-band VV configuration.

The profile length used during *in situ* roughness measurements has an important influence on the RMS height and correlation length parameterization. Shorter profiles result in lower RMS heights and correlation lengths and lead to over- or underestimation of the moisture content, depending on the roughness of the surface and the sensor configuration. On the other hand, longer profiles give rise to higher roughness parameters with reduced variability, and consequently, result in more stable retrieval results. However, the exact spatial scale at which roughness needs to be measured in order to describe the scattering on rough surfaces is not yet known.

The number of profiles over which RMS height and correlation length are averaged only has a moderate impact on the final roughness parameters and soil moisture retrieval results. Generally, a higher number of profiles is required for shorter profiles, surfaces with higher correlation lengths, higher soil moisture contents, and L-band HH configuration.

The horizontal spacing between height points measured along a profile is of low influence on the soil moisture retrieval, yet may cause confusion in the determination of the appropriate ACF. A misinterpretation of the slope of the ACF can lead to errors covering the complete range of moisture content.

Instrument inaccuracies up to ±2 mm, typically found for most current instruments, have a negligible impact on the soil moisture retrieval result. However, inaccuracies of ±5 mm may lead to errors ranging from ±0.5 vol% up to ±8 vol%. Such inaccuracies are possible for roughness parameterized using a meshboard and manual digitization.

Probably the most prevailing aspect in the parameterization of roughness is the removal of surface trends. In case the surface is characterized by an undulating trend, a linear removal may lead to retrieval errors up to 25 vol%, as was found on 4-m profiles of (*s,l*) = (1 cm,5 cm) with added cosine function. More precise retrieval results are obtained through the removal of a third-order polynomial, with errors less than 7.5 vol% irrespective of the type of trend and sensor configuration used. Nevertheless, further research needs to explore more complex detrending techniques and evaluate the retrieval errors involved.

As shown in this paper, the parameterization of surface roughness is not obvious. In the demonstrated experiments, various aspects were treated individually. However, in practice, different parameterization problems show up simultaneously, through which roughness errors may add up or cancel out. Therefore, future research definitely needs to clarify the errors involved in the entire parameterization and soil moisture retrieval process. Moreover, optimal standard parameterization procedures should be developed in function of the sensor configuration and target properties under study. Finally, being a theoretical study, the obtained results should be corraborated with experimental SAR observations.

## Figures and Tables

**Table 1.** Input parameters used for the IEM and the four-component dielectric mixing model.

**Model**	**Parameter**	**Value**
**Integral Equation Model**		
ENVISAT ASAR configuration	Frequency	5.3 GHz (C-band)
	Polarization	HH or VV
	Incidence angle	23°
ALOS PALSAR configuration	Frequency	1.27 GHz (L-band)
	Polarization	HH or VV
	Incidence angle	34.3°

**4-Component Dielectric Mixing Model**	Bulk density	1.2 g/cm^3^
	Specific density	2.65 g/cm^3^
	Sand content	15%
	Clay content	11.4%
	Temperature	15°C

**Table 2.** Average values of *s* and *l* obtained for different horizontal spacings. Standard deviations are added between brackets.

	Sample spacing				
	‘Truth’ (1 mm)	2mm	5mm	10mm	15mm
*s* (cm)	1	0.99 (0.00)	0.98 (0.00)	0.97 (0.01)	0.96 (0.02)
*l* (cm)	5	5.02 (0.04)	5.15 (0.11)	5.34 (0.22)	5.47 (0.40)

*s* (cm)	1	1.00 (0.00)	1.00 (0.00)	1.00 (0.00)	0.99 (0.00)
*l* (cm)	40	40.02 (0.04)	40.07 (0.21)	40.20 (0.41)	40.23 (0.37)

*s* (cm)	2	1.99 (0.01)	1.97 (0.01)	1.93 (0.02)	1.91 (0.01)
*l* (cm)	5	5.07 (0.05)	5.17 (0.08)	5.43 (0.19)	5.76 (0.52)

*s* (cm)	2	2.00 (0.00)	2.00 (0.00)	2.00 (0.01)	1.99 (0.01)
*l* (cm)	40	40.03 (0.05)	40.16 (0.28)	40.30 (0.45)	40.26 (0.58)

**Table 3.** RMSE values of *s* and *l* for different values of instrument accuracy.

Correct (*s,l*)	RMSE on *s* or *l* due to					
	1mm noise		2mm noise		5mm noise	
	*s* (cm)	*l* (cm)	*s* (cm)	*l* (cm)	*s* (cm)	*l* (cm)
(1 cm,5 cm)	0.0019	0.0316	0.0074	0.0775	0.0443	0.3782
(1 cm,40 cm)	0.0017	0.1673	0.0068	0.4764	0.0421	2.9972
(2 cm,5 cm)	0.0015	0.0447	0.0043	0.0316	0.0215	0.1265
(2 cm,40 cm)	0.0009	0.0707	0.0035	0.1871	0.0219	0.9301

**Table 4.** RMSE values of the retrieved soil moisture contents due to roughness parameterization errors, introduced by instrument noise.

(*s,l*) of original profile	Soil moisture content (vol%)	RMSE (vol%) of retrieved soil moisture due to		
		1 mm noise	2 mm noise	5 mm noise
(1 cm,5 cm)	5	0.04	0.11	0.54
(1 cm,5 cm)	15	0.11	0.30	1.39
(1 cm,5 cm)	25	0.21	0.57	2.61
(1 cm,5 cm)	35	0.34	0.92	4.15

(1 cm,40 cm)	35	0.39	2.08	8.31
(2 cm,5 cm)	35	0.24	0.26	0.51
(2 cm,40 cm)	35	0.23	0.46	2.76

**Table 5.** Average values of *s* and *l*, calculated after detrending of 4-m profiles. Standard deviations are added between brackets.

	**Correct roughness values**							

	*s* (cm)	*l* (cm)	*s* (cm)	*l* (cm)	*s* (cm)	*l* (cm)	*s* (cm)	*l* (cm)

	1	5	1	40	2	5	2	40
**Detrending type**	**Linear trended surface**							

Linear 4-m	0.99 (0.01)	4.93 (0.13)	0.91 (0.07)	30.09 (7.48)	1.97 (0.03)	4.76 (0.25)	1.80 (0.18)	31.57 (6.00)
Piecewise 1-m	0.92 (0.03)	3.80 (0.45)	0.51 (0.04)	8.31 (1.34)	1.85 (0.06)	3.77 (0.40)	0.98 (0.12)	7.45 (1.12)
Second-order	0.98 (0.02)	4.67 (0.29)	0.81 (0.10)	24.22 (7.40)	1.94 (0.04)	4.51 (0.26)	1.57 (0.19)	22.00 (7.58)
Third-order	0.96 (0.02)	4.36 (0.30)	0.64 (0.05)	15.02 (3.96)	1.93 (0.03)	4.37 (0.26)	1.41 (0.16)	17.84 (6.18)

	**Cosine trended surface**							

Linear 4-m	2.64 (0.13)	50.07 (2.25)	2.78 (0.42)	54.30 (4.68)	3.07 (0.26)	28.13 (8.31)	3.01 (0.74)	44.81 (7.06)
Piecewise 1-m	0.95 (0.04)	4.13 (0.49)	0.58 (0.06)	9.40 (1.47)	1.85 (0.05)	3.79 (0.36)	1.00 (0.10)	7.79 (1.47)
Second-order	1.32 (0.13)	13.48 (6.67)	1.10 (0.35)	25.47 (8.16)	2.17 (0.13)	7.19 (2.03)	1.88 (0.27)	28.38 (8.27)
Third-order	1.04 (0.04)	5.33 (0.53)	0.79 (0.12)	18.16 (4.72)	1.95 (0.05)	4.53 (0.30)	1.47 (0.12)	18.81 (5.13)
